# Management of Cardiac Involvement in Becker Muscular Dystrophy: A Case Report

**DOI:** 10.7759/cureus.73029

**Published:** 2024-11-05

**Authors:** Taulant Gishto, Silvia Methoxha, Naltin Shuka, Rudina Preci, Leonard Simoni

**Affiliations:** 1 Cardiovascular Disease, University Hospital Center "Mother Teresa", Tirana, ALB; 2 Cardiovascular Medicine, University Hospital Center "Mother Teresa", Tirana, ALB

**Keywords:** becker muscular dystrophy, cardiac involvement, dilated-cardiomyopathy, heart failure with reduced ejection fraction, implantable cardioverter-defibrillator (icd)

## Abstract

Becker muscular dystrophy (BMD) is an X-linked recessive neuromuscular disorder caused by a mutation in the dystrophin gene. Cardiac involvement is a frequent finding in BMD, and manifestations may vary from asymptomatic cardiac involvement to developing symptoms of heart failure and severe cardiomyopathy. We presented the case of a 32-year-old wheelchair-dependent BMD patient who came to our cardiology clinic with a two-month history of heart palpitations, rest and nocturnal dyspnea, fatigue, and generalized muscular weakness. Upon evaluation, a 24-hour Holter rhythm showed complex ventricular arrhythmia and 300 polymorphic ventricular extrasystoles with episodes of ventricular bigeminy, while echocardiography revealed a dilated left ventricle with severe systolic dysfunction (left ventricular ejection fraction (LVEF) 23%) and impaired global contractility. An implantable cardioverter defibrillator (ICD) was implanted, and guideline direct medical therapy (GDMT), sacubitril/valsartan, bisoprolol, furosemide, spironolactone, and dapagliflozin were initiated. The patient was discharged five days later, in an improved clinical condition, without dyspnea. A follow-up appointment two weeks after discharge was recommended in order to evaluate the patient's symptoms and the effectiveness of GDMT and a follow-up echocardiography at least three months after discharge to evaluate the heart's systolic and diastolic function.

## Introduction

Becker muscular dystrophy (BMD) is an X-linked recessive disorder characterized by mutations in the dystrophin gene located on chromosome Xp21.1, which codes for the central protein in the dystrophin-glycoprotein complex in skeletal and heart muscle cells. This complex has mechanical stabilizing and signaling roles in mediating interactions between the cytoskeleton, membrane, and extracellular matrix. Loss of function of this complex results in instability of the plasma membrane and myofiber loss in both skeletal and cardiac myocytes, resulting in progressive muscular weakness and degeneration, but also cardiac involvement that may lead to dilated cardiomyopathy [1,2].

The global prevalence of BMD is estimated to be 1.6 per 100,000 people [3]. BMD is considered a milder form of Duchenne muscular dystrophy, with heterogeneous clinical presentation and onset of symptoms varying between five and 60 years old [4].

Asymptomatic elevated CK (creatine kinase) levels, proximal lower limb girdle hyposthenia, myalgia, and muscular cramps are the most usual form of initial presentation in BMD patients. Muscle pseudo-hypertrophy due to the fibrosis and fatty replacement of atrophic muscles is a classical feature of BMD [5].

Cardiac involvement is seen in 60-75% of BMD cases, with an average onset of 28.7 ± 7.1 years [6]. Cardiac manifestations may vary from asymptomatic cardiac involvement to developing symptoms of heart failure and severe cardiomyopathy, while a correlation between skeletal muscle involvement and the onset time or severity of the cardiac involvement is not established. Cardiac involvement may also be the predominant manifestation with no evidence of muscular disease [7].

## Case presentation

A 32-year-old male presented to our cardiology clinic complaining of a two-month history of palpitations, paroxysmal nocturnal dyspnea, and generalized fatigue. The patient received a diagnosis of Becker muscular dystrophy at a young age, and at age 23, he began experiencing posture and gait disturbances, as well as lower limb weakness, which left him wheelchair-bound.

Molecular analysis of the dystrophin gene, undertaken in 2009, showed deletion of exons 5-6-7-8, confirming the diagnosis of Becker muscular dystrophy.

**Figure 1:**
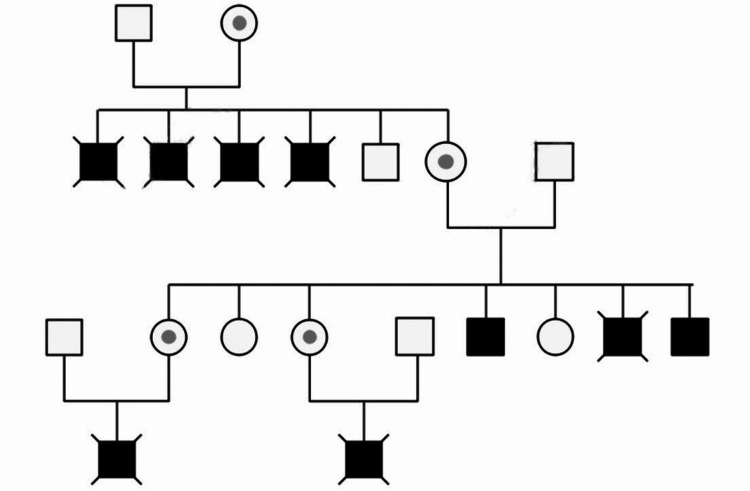
Pedigree chart circle: female; square: male; dotted circle: female carrier; black filled square: affected male; crossed black filled square: deceased affected male

The patient had a family history of muscular dystrophy. His maternal great-great-grandmother had lower limb muscular weakness and calf hypertrophy. Four of his five uncles were diagnosed with muscular dystrophy and passed away at a young age. From his parents' marriage, seven children were born, consisting of four females and three males. All three males were affected by BMD. His first brother began experiencing symptoms at the age of eight and became wheelchair-bound at the age of 26. His second brother also began experiencing symptoms at the age of eight, became wheelchair dependent at the age of 17, and passed away due to pneumonia at the age of 36. Two of his sisters were carriers, and they each had a son. One of them passed away at the age of 23 because of cardiac arrest, and the other was alive but wheelchair dependent at the time of presentation (Figure 1).

On physical examination, the patient was alert, with no cognitive deficits. Auscultation revealed a grade II systolic murmur at the apex, radiating to the axilla, and a normal vesicular breath sound without crackles. The neurological exam revealed paraplegia in the lower limbs, hypertrophy of the calves, hypotrophy of the quadriceps, a negative deep tendon reflex, and paraparesis in the upper limbs.

**Figure 2:**
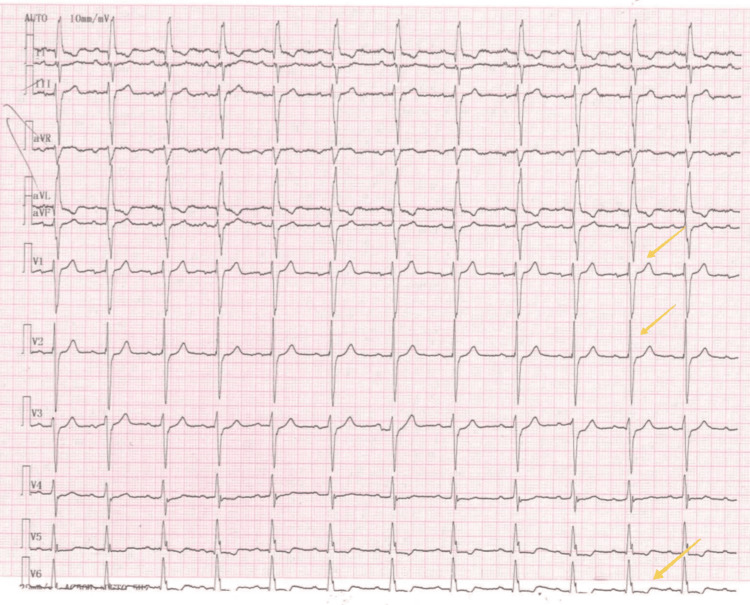
Resting ECG of the patient upon admission Arrow: showing tall R-waves in right leads and strain pattern (with ST depression and T-wave inversion in D1, aVL, V5-V6)

His resting ECG showed sinus rhythm, with a heart rate of ~75 bpm, strain pattern, and tall R waves in the right leads (Figure 2).

Upon admission, his complete blood count was unremarkable, while his biochemistry panel was altered, respectively: creatine kinase (CK) 2300 U/L, creatine kinase-myoglobin binding (CK-MB) 19.7 ng/mL, alanine transaminase (ALT) 69 U/L, aspartate aminotransferase (AST) 45 U/L, total cholesterol 226 mg/dL, triglycerides 577 mg/dL (indicating myocyte degeneration), and N-terminal pro-b-type natriuretic peptide (NT-proBNP) 1032 pg/mL (indicating heart failure). Laboratory investigations and reference values are shown in Table 1. 

**Table 1:** Complete blood count and the biochemistry panel WBC: white blood cells; NEU: neutrophils; LYM: lymphocytes; RBC: red blood cells; HB: hemoglobin; HCT: hematocrit; PLT: platelets; Creat: creatinine; Na: sodium; K: potassium; Cl: chloride; CRP: C-reactive protein; Tot bilirubin: total bilirubin; AST: aspartate aminotransferase; ALT: alanine transaminase; CK: creatine kinase; CK-MB: creatine kinase-myoglobin binding; NTproBNP: N-terminal pro-b-type natriuretic peptide; TG: triglycerides

Complete Blood Count			
Parameters	Reference range	Units	Patient’s values
RBC	4-5.5	X 10^6^/uL	4.2
HCT	36-52	%	38.79
HB	12-16.5	g/dL	13.7
WBC	4-10.5	K/uL	5.23
PLT	150-400	K/uL	258
Biochemistry Panel			
Urea	21-43	mg/dL	29.1
Creat	0.57-1.11	mg/dL	0.41
Na	136-145	mmol/L	137
K	3.5-5.1	mmol/L	3.9
Cl	98-107	mmol/L	98
Tot Bilirubin	0.3-1.2	mg/dL	0.4
ALT-SGPT	<55	U/L	69
AST-SGOT	5-34	U/L	45
CK	29-168	U/L	2300
CK-MB	<5.2	ng/mL	19.7
Troponin-I	<0.016	ng/mL	0.016
Tot Cholesterol	45-220	mg/dL	226
LDL- chol	<130	mg/dL	54
TG	50-180	mg/dL	577
CRP	<0.5	mg/dL	0.3
Glucose	82-115	mg/dL	91
NT-proBNP	<125	pg/mL	1032

A 24-hour Holter rhythm showed predominantly sinus rhythm, with an average heart rate of 81 bpm, a minimum heart rate of 64 bpm, and a maximum heart rate of 118 bpm. The patient displayed 300 polymorphic ventricular extrasystoles, accompanied by episodes of ventricular bigeminy. No significant pauses over 2.5 sec (significant reference range >3 sec).

Cardiac echography showed a dilated left ventricle with severe systolic dysfunction and impaired global contractility; Grade II: pseudonormal diastolic dysfunction; moderate mitral regurgitation (related to left ventricle dilation); mild tricuspid regurgitation; and no present pericardial fluid.

**Figure 3:**
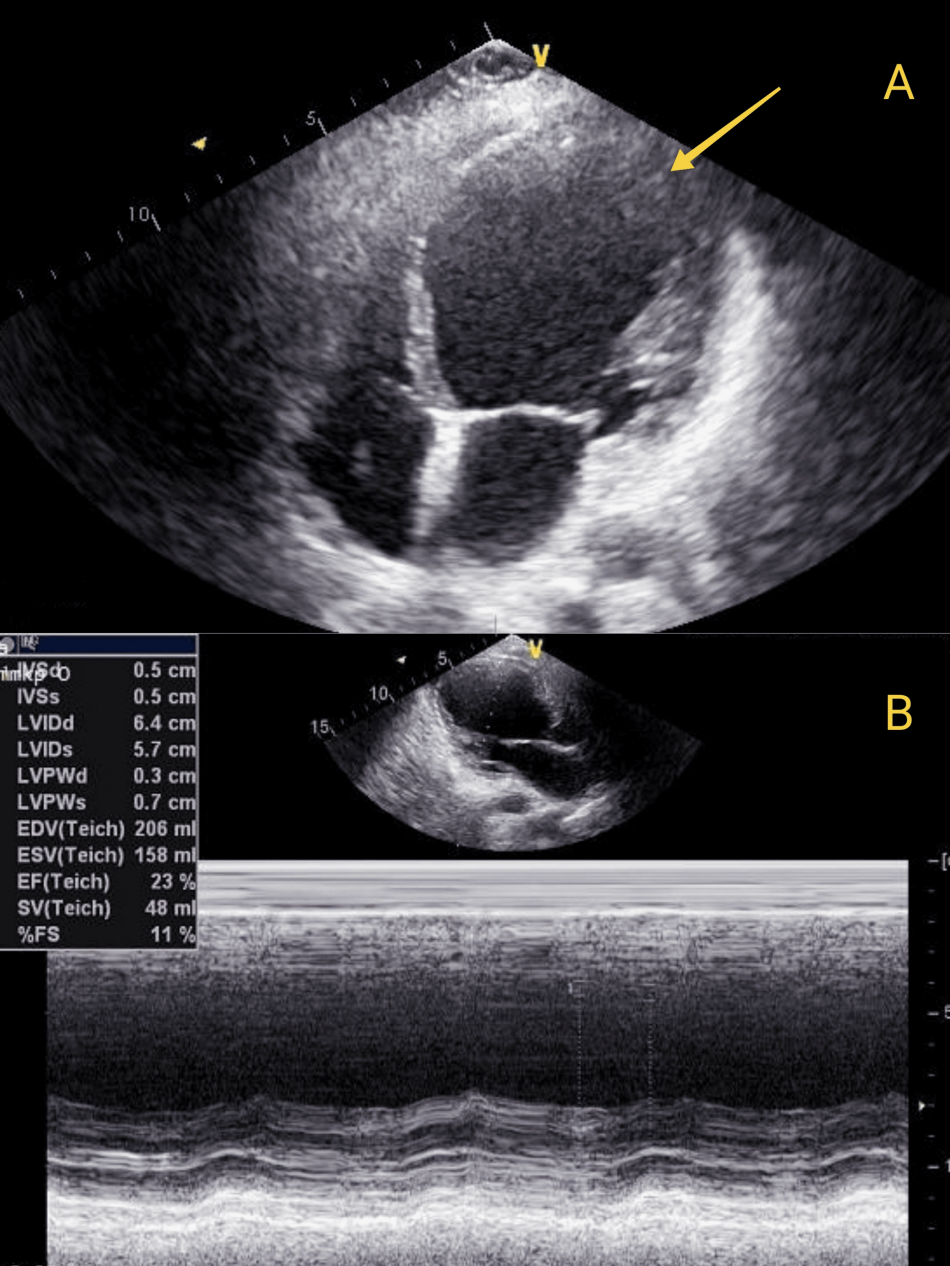
Cardiac echography A: apical 4 chamber view (arrow: dilated left ventricle); B: M-mode left ventricle measurements

Left ventricular dysfunction (LVD)=64mm (ref range 37-56mm), left ventricular systolic function (LVs)=57mm (ref range 22-38mm), left ventricle ejection fraction (LVEF)=23%, FS%=11%) (Figure 3).

It was decided by our cardiology team that due to complex ventricular arrhythmia (the presence of multiple polymorphic ventricular extrasystoles and ventricular bigeminy) accompanied by dilated cardiomyopathy, an implantable cardioverter defibrillator (ICD) should be implanted in order to be able to avoid fatal malignant arrhythmias such as ventricular fibrillation and sudden cardiac death.

A Medtronic-Mirro MRI DR SureScan DDME3D4 (Medtronic, Inc., Minneapolis, Minnesota, US) device was implanted. Dual chamber pacing was chosen due to higher diagnostic accuracy and due to supraventricular tachycardia (SVT), and VT discriminators that are not available in single-chamber ICDs. The procedure was performed without complications.

Upon admission, guideline-directed medical therapy (GDMT) was also initiated with sacubitril/valsartan (2x50 mg/day), furosemide (2x20 mg/day), spironolactone (25 mg/day), dapagliflozin (10 mg/day), fenofibrate (160 mg/day), and bisoprolol (2.5 mg/day). After five days, the patient's condition improved, leading to the discharge with the same therapy. We recommended a follow-up appointment within two weeks post-discharge to monitor the patient's response to GDMT, and a follow-up echocardiography at least three months after discharge to evaluate the heart's systolic and diastolic function.

## Discussion

Apart from progressive proximal skeletal muscle weakness and wasting, BMD is characterized by cardiac muscle involvement. Cardiac involvement may be asymptomatic and detected by instrumental investigations, but it may also manifest as heart failure, which is seen to be the main cause of death in BMD patients [8].

Progressive replacement of dysfunctional and degeneration of cardiomyocytes, especially in the inferolateral wall and the electrical conduction system with adipose and fibrous tissue, is seen pathoanatomically in BMD patients [9].

According to the American Heart Association (AHA), all BMD patients should have an initial cardiac evaluation with examination, ECG, and imaging performed at diagnosis, such as transthoracic echocardiogram (TTE), or even advanced cardiac imaging such as CT or cardiac magnetic resonance (CMR) [10].

BMD patients with cardiac involvement may complain about palpitations, dizziness, syncope, dyspnea at rest or during exercise, leg edema, or coughing [11]. One-third of patients diagnosed with BMD develop dilated cardiomyopathy with concomitant heart failure symptoms [12]. Hypertrophic cardiomyopathy is also reported as a cardiac manifestation in Becker's by Hayashi et al. [13] and Park O Y et al. Severe hypertrophy of both the left and right ventricles was observed with preserved LVEF and in a very short time progression in dilated cardiomyopathy with low LVEF and global hypokinesia. [14]. This finding should not be overlooked, as it may be an early indicator of progression into DCM and heart failure.

Al-Raqad M K et al. [15] and Xiong et al. [16] concluded in their studies that more than 76% of BMD patients presented with ECG abnormalities, the first ECG changes occurring early in the course of the disease (from 12 to 17 years) and that abnormal ECG findings should be considered as an early alarming sign for early evolving dilated cardiomyopathy and often may require further diagnostic assessments.

Typical ECG findings include tall R waves in the right precordial leads, an R:S ratio ≥ 1 in lead V1, deep Q waves in the inferolateral leads, a short pulse rate (PR), and a longer QTc interval. Conduction abnormalities may be present in the form of a right bundle branch block and a left bundle branch block (complete or incomplete), infra-hisian block [17,8].

Tachyarrhythmias may occur after the development of dilated cardiomyopathy. Pacemakers or implantable cardioverter-defibrillators are considered for BMD patients based on guidelines used for non-ischemic cardiomyopathies [6].

Echocardiography may show myocardial thickening with normal cavity size and preserved systolic function in the case of hypertrophic cardiomyopathy, which tends to evolve into dilated cardiomyopathy, reported by Park O Y et al. [14]. Dilated cardiomyopathy in BMD manifests with dilated cardiac cavities, wall motion abnormalities, or global hypokinesia; isolated right or left ventricle dilation, or a combination of right and left wall abnormalities [18].

Cardiac magnetic resonance provides not only anatomical and functional information but also tissue characterization. BMD cardiomyopathy may present in CMR with left and right ventricular dilatation, hypertrophy, presence of fatty infiltration, diffuse or focal fibrosis, and ventricular dysfunction [19].

In neuromuscular dystrophies, in case of evidence of reduced left ventricular function, it is recommended to pursue guideline-directed heart failure management. Angiotensin-converting enzyme (ACE) inhibitors and angiotensin II receptor blockers (ARBs) and beta-blockers can improve ventricular function in patients treated early [20]. Further studies should be carried out to evaluate the improvement of overall heart function and prognosis in patients on GDMT with muscular dystrophies.

On average, patients with BMD survive into their mid-40s. The most common cause of death is heart failure due to cardiomyopathy [8].

## Conclusions

Becker muscular dystrophy with cardiac involvement is a rare finding in our daily clinical practice. Cardiac involvement in our patient manifested as mild heart failure symptoms with dilated cardiomyopathy and rhythm disturbances. Our cardiology team evaluated the patient's case and implemented a tailored approach, implanting an ICD and adhering to heart failure guidelines in the field of dilated cardiomyopathy. The patient’s clinical symptoms improved. A follow-up appointment two weeks after discharge was recommended in order to evaluate the patient's symptoms and the effectiveness of GDMT. At least three months after discharge, a follow-up echocardiography was conducted to assess the heart's systolic and diastolic function.

Multidisciplinary collaboration is very important in the management of rare conditions, such as in the case of BMD. Cooperation between cardiologists, arrhythmologists, advanced heart failure therapists, geneticists, and neurologists is a key link in the management of these patients. Genetic counseling may be considered for family members. Educating and informing the patient about the disease and the prognosis is of particular importance, as is the discussion with the patient about the long-term management plan and the possibility of interventions in the future. More studies are necessary to better understand this condition and improve management strategies.
